# The effect of replacing sedentary behavior with different intensities of physical activity on depression and anxiety in Chinese university students: an isotemporal substitution model

**DOI:** 10.1186/s12889-024-18914-y

**Published:** 2024-05-23

**Authors:** Yulan Zhou, Zan Huang, Yanjie Liu, Dongao Liu

**Affiliations:** 1 College of Physical Education and Health Sciences, Zhejiang Normal University, Jinhua, Zhejiang 321004 China; 2 Physical Education Department, Shanghai University of Finance and Economics, Shanghai, 200433 China

**Keywords:** Physical activity, Sedentary, Depression, Anxiety, University students

## Abstract

**Background:**

Previous research has suggested that engaging in regular physical activity (PA) can help to reduce symptoms of depression and anxiety in university students. However, there is a lack of evidence regarding the impact of reducing sedentary behavior (SB) and increasing light-intensity PA (LPA) on these symptoms. This study aims to address this gap by using isotemporal substitution (IS) models to explore how substituting SB with LPA or moderate-to-vigorous PA (MVPA) affects depression and anxiety symptoms among university students.

**Methods:**

The study recruited 318 university students with a mean age of 21.13 years. Accelerometers were used to objectively measure the time spent on SB, LPA, and MVPA, while depression and anxiety symptoms were assessed using the Center for Epidemiologic Studies Depression Scale (CES-D) and the Self-rating Anxiety Scale (SAS). IS models using multivariable linear regression were employed to estimate the associations between different behaviors and depression and anxiety symptoms when 30 min of one behavior was substituted with another.

**Results:**

In the single-activity model, less SB (β = 0.321, 95% CI: 0.089, 1.297) and more MVPA (β = −0.142, 95% CI: −1.496, − 0.071) were found to be significantly and negatively associated with depression scores, while less SB (β = 0.343, 95% CI: 0.057, 1.014), LPA (β = 0.132, 95% CI: 0.049, 1.023), and more MVPA (β = −0.077, 95% CI: −1.446, − 0.052) were significantly and negatively correlated with anxiety scores. The IS analysis revealed that substituting 30 min of SB with LPA (β = −0.202, 95% CI: −1.371, − 0.146) or MVPA (β = −0.308, 95% CI: −0.970, − 0.073) was associated with improvements in depressive symptoms. Substituting 30 min of SB with MVPA (β = −0.147, 95% CI: −1.863, − 0.034) was associated with reduced anxiety symptoms.

**Conclusion:**

Replacing 30 min of SB with MVPA may alleviate depression and anxiety symptoms in university students. Further research is needed to explore the long-term effects of PA interventions on the mental health disorders of this population.

**Supplementary Information:**

The online version contains supplementary material available at 10.1186/s12889-024-18914-y.

## Background

The World Health Organization (WHO) estimates that people worldwide will be affected by mental health disorders at some point in their lives. Around one billion people currently suffer from such conditions, placing mental health disorders among the leading causes of disease burden and disability worldwide [1]. Furthermore, individuals with moderate to severe mental health disorders have a reduced life expectancy of 10–20 years and a 2–3 times higher risk of mortality compared to the general population [2]. Depression and anxiety are globally prevalent mental health disorders and increased by a massive 25% in the first year of the COVID-19 pandemic [1]. Both disorders are negative emotions and often co-occur, with 62% of adults with anxiety experiencing depressive episodes as well [3]. The pressures of academics, interpersonal interactions, and employment make university students particularly vulnerable to mental health disorders, including depression and anxiety [4]. Consequently, the annual detection rate of these conditions among university students is on the rise globally [5]. A survey conducted in China has revealed that a significant number of university students are at high risk of developing depression and anxiety. Specifically, 6.6% of students face a high risk of depression, while 5.4% exhibit severe anxiety disorders [6]. The presence of depression and anxiety during university can persist into adulthood and adversely affect many aspects of personal life, such as personal relationships, academic performance, and work productivity [7]. It is essential to address the mental health needs of university students who experience depression and anxiety.

Research has shown that engaging in regular physical activity (PA) can provide numerous health benefits for university students, including a reduced risk of depression and anxiety [8, 9]. Studies have found a strong connection between moderate-to-vigorous PA (MVPA) and lower levels of both depression and anxiety symptoms in this population. Two recent systematic reviews, which encompassed evidence from prospective cohort studies and intervention studies, have converged on the conclusion that engaging in regular MVPA is linked to a reduction in depressive and anxiety symptoms [10, 11]. Moreover, while the relationship between light-intensity PA (LPA) and sedentary behavior (SB) with depression and anxiety is not entirely conclusive, some studies have found links between these factors [12, 13]. Given the health-promoting relationship between PA and health, public health organizations worldwide encourage individuals to “sit less and move more” [14, 15]. However, individuals are limited in the amount of time they can engage in PA each day, and changes in the duration of one behavior inevitably led to compensatory changes in the duration of other behaviors. Therefore, a more comprehensive approach should be used to explore the combined effects of different intensities of PA and SB on health outcomes [16].

The Isotemporal Substitution (IS) Model as suggested by Mekary et al. [17] simultaneously simulates the specific activity being performed and the specific behavior being replaced in an equal time-exchange manner. The model controls for the confounding effect of total activity time and the heterogeneity of participation or substitution activities. Thus, one can estimate associations between theoretically substituting one type of PA for others and health outcomes. Several recent studies have explored the associations of SB, LPA, and MVPA with symptoms of depression and anxiety in older adults using the IS modeling method. For example, two cross-sectional studies have shown that reallocating 30 min of SB with an equal amount of either LPA or MVPA is significantly associated with a reduced risk of developing depression symptoms among older adults [18, 19]. The studies by Dillon et al. [20] and Tully et al. [21] demonstrated that reallocating 30 min of SB with LPA or MVPA was associated with improved anxiety symptoms among older adult. In a study by Chao et al. [22], Chinese university students experienced a noteworthy reduction in anxiety symptoms by replacing 15 min of SB with LPA. Nonetheless, the impact of substituting SB with various intensities of PA on depression among university students remains an area that requires further exploration. Moreover, unlike depression, anxiety often presents with distinct physiological symptoms such as a racing heart, muscle tension, sweaty palms, and dry mouth [23]. Given these distinctions, it becomes imperative to explore whether the substitution relationship between various activity behaviors differs in its effects on depression and anxiety among university students.

Therefore, this study aimed to investigate the cross-sectional associations between SB, LPA, and MVPA with depression and anxiety among university students, and to explore the difference in the effects of replacing 30 min of SB with different intensity PA (LPA and MVPA) on depression and anxiety among university students. The outcomes of this study hold the potential to enrich our comprehension of the intricate connection between PA and the prevalence of depression and anxiety among university students. Furthermore, they offer valuable practical insights that can inform the development of effective interventions aimed at promoting PA and mitigating these mental health disorders within this population.

## Methods

### Participants and data collection

For this study, participants were university students recruited via a convenience-based sampling method. Recruitment efforts were concentrated on one sizable public university in each of the regions: Hubei Province, Zhejiang Province, and Shanghai, China. A multistage cluster sampling approach was employed to select participants. In the first stage, one college (e.g., Humanities, social sciences, engineering, and information sciences) was chosen from each of the selected universities. Following that, two classes were selected from each of the selected colleges. To be eligible for participation in the study, individuals needed to meet the following criteria: They had to be full-time university students between the ages of 18 and 25 years old. Participants who reported any physical or mental condition that would hinder their ability to engage in PA were excluded from the study. Ethical approval from the Ethics Committee of Zhejiang Normal University was obtained before the commencement of data collection for our study. Informed consent was obtained from all participants before they completed the questionnaire. To ensure confidentiality, participants were assigned a unique identification number and all data collected were kept secure and anonymous. Participants were informed that they could withdraw from the study at any time without penalty. The study’s required number of participants was estimated using G*Power 3.1 software, considering a 5% maximum tolerable error and a power of 0.8. The estimated number of subjects needed was 343. To accommodate potential losses such as dropouts and hardware failures, this number was increased by 20%. Therefore, a total of 463 university students from 6 classes were invited to participate in the survey. Ten students declined to cooperate with the survey, and an additional seven students were excluded due to recent psychological dysfunction, defined as having received a psychological disorder diagnosis within the past 6 months. Consequently, a total of 446 students actively participated in this study. An initial inspection of the raw data showed that 104 participants did not provide valid accelerometry data (at least 10 h of wear per day was considered one valid day, and at least one valid weekend day and two valid weekdays), and a further 24 participants did not provide valid survey data for the outcome variables. Thus, the study included a total of 318 participants, with 107 from Hubei Province, 93 from Zhejiang Province, and 118 from Shanghai.

Data collection took place during the middle of the Fall semester, spanning from October to December 2022. The primary author, alongside two research assistants who were postgraduates specializing in physical education, conducted the data collection. Participants were equipped with accelerometers and instructed to maintain their regular daily routines during the monitoring period. To ensure adherence, the research assistants made daily visits to the universities in the mornings to remind students to wear the accelerometers. To ensure the collection of data for a complete seven days, students were instructed to return the accelerometers after eight days. Subsequently, all participants were requested to complete a self-administered questionnaire in a classroom environment. This questionnaire covered various socio-demographic factors (e.g., age and gender), lifestyle aspects (e.g., alcohol consumption and smoking habits), sleep patterns, and details regarding mental health disorders. Throughout this process, the research assistants were present to offer support to the participants and ensure order in the classrooms.

### Measurements

#### Sedentary behavior and physical activity

SB, LPA, and MVPA were measured using the triaxial accelerometer (ActiGraph wGT3X-BT). The technical reliability and validity of the accelerometer device have been described elsewhere [24]. Participants were instructed to wear the accelerometers on their right hipbone for at least seven consecutive days and only remove it for sleeping and water-based activities (e.g., swimming and bathing). The accelerometer started recording data at 0:00 a.m. on the second day of distribution and continued until the researcher retrieved it at the end of the eighth day. After the test was conducted, data were extracted using Actilife 6.5 software and then collapsed into a specific time interval (epoch), for example, a 60 s epoch. The inclusion criteria of wearing the accelerometer for at least one valid weekend day and two valid weekdays, with at least 10 h per day of wear, helps to ensure that the data is representative of the participants’ typical PA levels [25]. Non-wear time was defined as a period of at least consecutive 60 min during which the accelerometer recorded 0 counts per minute (cpm) [26]. Activity counts were classified using a set of cut points to calculate the intensity and amount of SB, LPA, and MVPA. SB was classified as < 100 cpm, LPA was 100–1952 cpm, and MVPA was > 1952 cpm [27].

#### Depression and anxiety

The Center for Epidemiologic Studies Depression (CES-D) 20-item symptom scale was used to assess symptoms of depression [28]. The CES-D is a widely used and well-established measure for assessing symptoms of depression in research studies [29]. Participants were asked to report how often over the past week they have experienced each of the 20 symptoms associated with depression such as restless sleep, poor appetite, and feeling lonely. The score of each item ranges from 0 (rarely or none of the time) to 3 (most or all of the time). The total score ranged from 0 to 60, with higher scores indicative of higher levels of depressive symptoms. A score of 16 points or more is indicative of depression in this assessment [30]. The reliability of the CES-D in this study, as indicated by a Cronbach’s alpha coefficient of 0.865, is well above the acceptable threshold of 0.70, indicating that the scale is consistent in measuring symptoms of depression.

The Self-rating Anxiety Scale (SAS), which was compiled by Zung et al. [31] was employed in this study to allow university students to self-report anxiety symptoms, which can provide insight into the subjective experience of anxiety. The survey consists of 20-item scale and covers a range of potential anxiety symptoms, including psychological and somatic symptoms. Each item is score on a four-point Likert scale according to the frequency of the status in the previous week. Participants choose responses ranging from 1 to 4 (1 = no or a little of the time, 2 = some of the time, 3 = good part of the time, 4 = most of the time or all the time) with summed scores ranging from 20 to 80. Higher scores indicate a higher level of anxiety symptoms. A cut-off value of 50 for the total score was established to indicate the presence of anxiety symptoms [7]. This scale has been shown to have good reliability and validity in a variety of populations [32]. In this study, the internal consistency of the scale was also found to be high, with a Cronbach’s alpha coefficient of 0.782, indicating that the scale is reliable in measuring this construct in the study population.

### Covariates

Covariates were selected based on previous studies and included socio-demographic characteristics (i.e., weight, height, age, gender, years of university, and residential background), lifestyle aspects (i.e., alcohol consumption and smoking information), and sleep pattern [33]. Participants were asked to report their socio-demographic and lifestyle information through questionnaires. The Pittsburgh sleep quality index (PSQI) questionnaire were used to assess students’ sleep patterns [34]. The researchers also calculated the participants’ body mass index (BMI) using their reported weight and height (weight in kilograms divided by height in meters squared).

### Data analysis

Statistical analyses were performed using IBM SPSS Statistics, Version 26.0 for Windows and the level of significance was set at *P* < 0.05. Descriptive statistics like frequencies, percentages, means, and standard deviations were used to summarize the data. Categorical variables like gender, drinking alcohol, and smoking status were presented as frequencies and percentages. Continuous variables like age and BMI were presented as means and standard deviations. Person correlations were used to assess the associations among SB, LPA, MVPA, and mental health disorders. Three multiple linear regression models including a single-activity, a partition, and an IS models were utilized to examine the relationship between SB, LPA, and MVPA with both depression and anxiety. Prior to conducting the analysis using three distinct linear regression models, it was ensured that there existed linear relationships between SB, LPA, MVPA, and the scores for depression and anxiety. Additionally, it was confirmed that there was no evidence of multicollinearity among the independent variables. In current study, we focused on modeling the effects of reallocating 30 min from one behavior to another. This approach was chosen for its practicality, especially considering that in China, university students tend to be generally physically inactive [30]. Reallocating 30 min is a more feasible and realistic scenario than longer durations. In addition, previous studies among adults have interpreted the association between replacing of 30 min with different activity intensities and mental health disorder [18–21]. We chose the replacing 30 min in the present study to improve the interpretability of the results. SB, LPA, and MVPA were standardized using 30 min as a unit for activity in analyses.

First, a series of single-activity models were computed to investigate the independent associations between each behavior (i.e., SB, LPA, MVPA) and mental health disorder (i.e., depression, anxiety), adjusted for covariates that are known to be associated with both activity and mental health disorder (e.g., age, gender, smoking status, and alcohol consumption). One type of single activity model (in the case of SB) is shown as follows: Mental health disorder = (β1) SB + (β5) covariates.

Second, partition models were used to estimate the effects of increasing each behavior on mental health disorder while holding the duration of each of the other behavior variables constant. Partition model represents the effects of adding, not substituting an activity type because total wear time is excluded in the model (thus is not held constant). Partition models were expressed as: Mental health disorder = (β1) SB + (β2) LPA + (β3) MVPA + (β5) covariates.

Finally, IS models were applied to explore the effects of reallocating time between SB, LPA, and MVPA on indicators of mental health disorder. IS models estimate the effects of replacing time spent engaging in one behavior with another behavior for the same amount of time, while holding total time constant. The following equation describes the effects of replacing 30 min of SB with 30 min of LPA (β2), or MVPA (β3): Mental health disorder = (β2) LPA + (β3) MVPA + (β4) total wear time + (β5) covariates. β1-β5 are the coefficients of respective activities or covariates.

## Results

### Descriptive characteristics of study sample

Table 1 shows the characteristics of study participants. The study included a final sample of 318 participants, of which 127 (39.9%) were male and 191 (60.1%) were female. The mean age was 21.13 (SD = 3.53) years. The mean BMI and total PSQI score were 19.48 (SD = 1.03) and 6.93 (SD = 2.13), respectively. On average, participants wore accelerometers for 823.89 (SD = 111.75) minutes/day. The mean proportion of SB, LPA, and MVPA time to total accelerometer wearing time were 72.5%, 21.5%, and 6.0%, respectively. Participants reported an average score of 13.85 (SD = 8.21) for depressive symptoms, with 17.3% of participants falling into the category of experiencing depressive symptoms (a total score of CES-D ≥ 16). In terms of anxiety symptoms, the mean score was 39.03 (SD = 6.20), and 26.1% of the sample met the criteria for anxiety symptoms (a total score of SAS ≥ 50). The correlation among SB, LPA, MVPA, and mental health problems presented in the supplemental Table 1.

**Table 1:** Characteristics of study participants

Variables	Total sample n (%) or Mean (SD)	
Gender		
Male	127	39.9%
Female	191	60.1%
Age (years)	21.13	3.53
Body mass index (kg/m^2^)	19.48	1.03
PSQI score	6.93	2.13
Years of university		
Freshman (Junior)	91	28.6%
Senior	227	71.4%
Residence		
Urban	192	60.4%
Rural	126	39.6%
Drinking alcohol status		
Yes	157	49.4%
No	161	50.6%
Smoking status		
Yes	8	2.5%
No	310	97.5%
Activity behavior (min/day)		
Total wear time	823.89	111.75
SB	597.00	88.50
LPA	177.16	50.36
MVPA	49.74	28.94
CES-D depression score	13.85	8.21
Normal (< 16)	263	82.7%
Caseness (≥ 16)	55	17.3%
SAS anxiety score	39.03	6.20
Normal (< 50)	235	73.9%
Caseness (≥ 50)	83	26.1%

*Note* Data are given as the number (%) or mean ± SD

*Abbreviation* PSQI, Pittsburgh Sleep Quality Index; SB, Sedentary behavior; LPA, Light-intensity physical activity; MVPA, Moderate-to-vigorous physical activity; CES-D, Center for Epidemiologic Studies Depression; SAS, Self-rating Anxiety Scale

### Effects of reallocating time between the different intensities of PA and SB on depression symptoms

Table 2 displays single-activity, partition, and IS models for the relationship between different intensities of PA, SB, and university students’ scores of depressive symptoms. In the single-activity models, SB time tended to be significantly and positively associated with depression scores (β = 0.321, 95% CI: 0.089 to 1.297), whereas MVPA was significantly and negatively associated with scores of depressive symptoms (β = −0.142, 95% CI: −1.496 to − 0.071). In the partition models, increasing SB by 30 min while holding the other variables constant was associated with a significant increase in depression scores among university students (β = 0.326, 95% CI: 0.098 to 1.315). In the IS models, replacing 30 min/day of SB with LPA (β = −0.202, 95% CI: −1.371 to − 0.146) and MVPA (β = −0.308, 95% CI: −0.970 to − 0.073) resulted in a significant decrease in depression scores.

**Table 2:** The associations of SB, LPA, and MVPA with depression score

	SB		LPA		MVPA	
	β	95%CI	β	95%CI	β	95%CI
Single-activity model	0.321^*^	(0.089, 1.297)	−0.084	(− 1.145, 0.323)	−0.142^*^	(− 1.496, − 0.071)
Partition model	0.326^*^	(0.098, 1.315)	−0.123	(− 1.305, 0.102)	−0.096	(− 2.051, 0.421)
Isotemporal model						
Replace SB with	Dropped		−0.202^*^	(− 1.371, − 0.146)	−0.308^*^	(− 0.970, − 0.073)
Replace LPA with	0.202^*^	(0.146, 1.371)	Dropped		−0.025	(− 1.647, 1.249)
Replace MVPA with	0.308^*^	(0.073, 0.970)	0.025	(− 1.249, 1.647)	Dropped	

*Note* All models adjusted for covariates of age, gender, BMI, sleep patterns, years of university, residential background, smoking status, and alcohol consumption. Substitution model adjusted for all covariates including total accelerometer wear time

β = Regression coefficients correspond to a 30 min increment of each activity

*Abbreviations* SB, Sedentary behavior; LPA, Light-intensity physical activity; MVPA, Moderate to vigorous physical activity

*, *P* ≤ 0.05

### Effects of reallocating time between the different intensities of PA and SB on anxiety symptoms

Table 3 presents the results for the single-activity, partition, and IS models adjusted for covariates. The single-activity model shows that higher levels of both SB (β = 0.343, 95% CI: 0.057 to 1.014) and LPA (β = 0.132, 95% CI: 0.049 to 1.023) were significantly associated with higher anxiety scores. Conversely, a higher level of MVPA was associated with a lower anxiety score (β = −0.077, 95% CI: −1.446 to − 0.052). The partition model showed that increasing SB by 30 min was associated with higher symptoms of anxiety (β = 0.325, 95% CI: 0.085 to 0.983). The IS model demonstrated that a 30 min unit of SB replaced with MVPA was significantly and negatively associated with anxiety scores (β = −0.147, 95% CI: −1.863 to − 0.034). No statistically significant change in scores of anxiety symptoms was observed when SB was substituted by LPA (β = −0.095, 95% CI: −0.982 to 0.281).

**Table 3:** The associations of SB, LPA, and MVPA with anxiety score

	SB		LPA		MVPA	
	β	95%CI	β	95%CI	β	95%CI
Single-activity model	0.343^*^	(0.057, 1.014)	0.132^*^	(0.049, 1.023)	−0.077^*^	(− 1.446, − 0.052)
Partition model	0.325^*^	(0.085, 0.983)	0.090	(− 0.181, 0.849)	−0.041	(− 1.169, 0.641)
Isotemporal model						
Replace SB with	Dropped		−0.095	(− 0.982, 0.281)	−0.147^*^	(− 1.863, − 0.034)
Replace LPA with	0.095	(− 0.281, 0.982)	Dropped		−0.093	(− 1.669, 0.471)
Replace MVPA with	0.147^*^	(0.034, 1.863)	0.093	(− 0.471, 1.669)	Dropped	

*Note* All models adjusted for covariates of age, gender, BMI, sleep patterns, years of university, residential background, smoking status, and alcohol consumption. Substitution model adjusted for all covariates including total accelerometer wear time

β = Regression coefficients correspond to a 30 min increment of each activity

*Abbreviations* SB, sedentary behavior; LPA, low-intensity physical activity; MVPA, moderate to vigorous physical activity

*, *P* ≤ 0.05

## Discussion

In the current study, the prevalence rates of depression and anxiety among university students were determined to be 17.3% and 26.1%, respectively. These findings align with surveys conducted among university students in various other countries [35, 36]. This highlights that depression and anxiety are major mental health concerns not confined to Chinese students but prevalent among university students worldwide [37, 38]. Furthermore, university students face unique challenges in terms of PA and SB. They often have demanding schedules and spend long periods of time sitting in lectures or studying. A comprehensive body of evidence has found PA to reduce depression and anxiety in both clinical and non-clinical populations [39, 40]. However, the beneficial effects of LPA and MVPA, as well as the impact of substituting SB with light activity or MVPA on mental health disorders, are less known. The IS model is likely to show more accurate results of associations of SB and PA with mental health disorders, since it takes the finite amount of time in a day into account, allowing for estimating the effect of replacing one type of PA with another. This study demonstrated the usefulness of the IS model approach in examining the relationship between PA and mental health disorders in Chinese university students. By estimating the effects of substituting SB with different intensities of PA, the study found that replacing 30 min of SB with MVPA was associated with decreased depression and anxiety scores. Additionally, replacing 30 min of SB with LPA was associated with lower depression scores.

The available evidence, specifically within the domain of IS modeling, is notably limited when it comes to addressing depressive symptoms among university students in comparison to studies conducted on other age demographics. Consistent with two cross-sectional studies among older adults [18, 19], the current study found a significant decrease in depressive symptoms when 30 min of SB was substituted with LPA or MVPA. These results suggest that the benefits of PA on depressive symptoms are not limited to older adults and can also be observed in younger populations. A larger number of intervention studies have also confirmed that PA can significantly reduce depressive symptoms [41]. Multiple mechanisms of action have been proposed to explain associations between PA and depressive symptoms. Depression is a negative mood that can be impacts how people think, feel, and go about daily activities. Typical symptoms of depression include sadness, emptiness, hopelessness, feeling of worth-lessness, and loss of interest in activities [42, 43]. Compared with SB, LPA or MVPA can increase the production and release of mood-related neurotransmitters such as serotonin and endorphins, which can help promote pleasure and positive feeling, thereby alleviating depressive symptoms [44, 45]. In addition, participation in PA can lead to improved social relationships and increased social support, which can help reduce psychological stress and improve depressive symptoms [46, 47]. Our study further revealed that compared to replacing 30 min of SB with LPA (− 0.202), a more substantial benefit was observed when replacing 30 min of SB with MVPA (− 0.308) concerning depressive symptoms in university students. A recent systematic review of IS studies also demonstrated that the strongest association with health outcomes is observed when time is reallocated from SB to MVPA [48]. These results imply that university students who spent most of their day sedentary (72.5%) should be encouraged to sit less and move more for a range of health benefits, including improvements in mental and cardiovascular health and reduced risk of chronic diseases. Furthermore, incorporating reduced SB and increased MVPA into daily life may be a more effective strategy for improving depressive symptoms in university students.

The findings derived from this study emphasized the positive consequences of replacing 30 min of SB with MVPA in mitigating anxiety among university students. Conversely, there was no observable effect when substituting 30 min of SB with LPA, marking a distinction from the ameliorative impact of LPA on depression. Anxiety is characterized as a distinct, unpleasant emotional state or condition encompassing apprehension, tension, worry, and physiological arousal [49]. It is essential to note that anxiety and depression represent two distinct and valid constructs that frequently occur simultaneously. Alternatively, they could be regarded as different expressions of the same underlying vulnerability [23]. In the model formulated by Clark et al. [50] symptoms of depression and anxiety are classified into three subtypes: negative affectivity, positive affectivity, and physiological hyperarousal. Negative affectivity is linked to both depression and anxiety. The deficiency in positive affectivity is hypothesized to be solely connected to depression, while physiological hyperarousal is suggested to be specific to anxiety. Consequently, consistent with the approach for addressing depression, substituting SB with PA holds promise for ameliorating the negative emotional dimensions of both depression and anxiety. This is believed to occur through the modulation of neuroplasticity and the reduction of inflammation [47, 51]. However, it’s worth noting that LPA may not be as effective in attenuating the physiological hyperarousal associated with anxiety. In contrast, engaging in a moderate or high level of PA has the potential to induce relaxation in the central nervous response and decrease the sensitivity of physiological arousal tied to anxiety, ultimately resulting in a reduction in anxiety [52]. Moreover, PA serves as a valuable form of distraction from the daily stressors that individuals encounter. Conversely, engaging in LPA may curtail students’ ability to divert their attention away from the stress-inducing factors of everyday life, leading to an escalation in the severity of anxiety symptoms [53].

Consistent with the established literature, current study provided further evidence that replacing SB with either LPA or MVPA yielded favorable effects on the depressive symptoms of participants [18, 19]. However, it’s important to emphasize that our investigate did not uncover any beneficial effects of LPA substituting SB on anxiety among university students. This outcome stands in contrast to the findings of studies conducted by Dillon et al. [20] and Chao et al. [22]. The inconsistent findings may be related to the use of different measures of physical behavior in these studies. Dillon et al. [20] used objective measures of physical behavior, the GENEActiv. This accelerometer measures acceleration at the wrist, while the ActiGraph, which used in present study, measures acceleration of the body at the hip. The movement or acceleration of the body differs significantly at these two positions and thus affect the comparability of the current findings to previous research. In an investigation led by Chao et al. [22], the central focus was on examining the connection between self-reported PA and anxiety among college students. It’s worth noting that when PA levels are assessed using self-reported measurement tools, there is a tendency for individuals to overestimate their activity levels [54]. This could offer an explanation for why previous research often demonstrated positive associations between LPA and anxiety, while our current study did not. Additionally, it is important to consider the context of LPA when examining its relationship with anxiety. Different contexts of LPA may have different effects on anxiety levels due to factors such as the level of mental stimulation they provide and the social context in which they are performed [55]. For example, household and occupational LPA may reduce anxiety by providing a sense of accomplishment and control, while transport LPA may be associated with anxiety due to the stress and time pressure involved [56]. The single model of this study also demonstrated that a more time spent in LPA was associated with higher anxiety scores. It is premature to entirely negate the potential effects of replacing SB with LPA. To gain a more thorough understanding of whether and to what extent LPA can be beneficial for the mental health of university students, additional research utilizing comprehensive measurement tools is essential.

Given the escalating prominence of depression and anxiety as significant public health concerns, it is paramount that we pinpoint cost-effective strategies to address these challenges. Our findings indicate that replacing 30 min of SB with LPA (β = −0.202, 95% CI: −1.371, − 0.146) or MVPA ((β = −0.308, 95% CI: −0.970, − 0.073) significantly improved depression symptoms, while only 30 min of MVPA (β = −0.147, 95% CI: −1.863, − 0.034) substitution for SB was effective in reducing anxiety symptoms among university students. Although the relatively small β coefficients and wide confidence intervals may indicate that the actual effect size is insufficient to confidently assert that such behavior substitution has a substantive improvement in university students’ depression and anxiety symptoms, the findings offer insights into optimizing PA implementation and highlight the challenges one may encounter in making such changes. For university students who are relatively physically robust, targeting substituting SB with MVPA may be a more feasible, attractive, or realistic behavior change to target in the first instance. However, for students who are not used to regular PA, attempting to switch from SB to MVPA may be too daunting and overwhelming. Encouraging students to find PA that they enjoy and can easily incorporate into their daily routine is key. Embarking on the journey with smaller, realistic goals can be instrumental in building both confidence and motivation. Following this, it is prudent to undergo exercise testing to tailor a PA program that aligns with these goals, preventing an initial overexertion. The use of an activity diary is strongly encouraged, and documenting daily life PA can enhance students’ commitment to the PA program. Finally, maintaining social connections, whether with parents or classmates, while engaging in PA can be a valuable factor in facilitating students’ achievement or maintenance of this new behavior [57, 58]. Furthermore, Ministries of Education and Health should place a strong emphasis on heightening public health awareness concerning the pivotal role of MVPA for individuals with mental health disorders. They should offer guidance on indispensable preventive measures for university students who are beginning to adopt a lifestyle of physical inactivity. Additionally, these ministries should actively adopt and implement effective policies and interventions related to these pertinent issues.

There are several potential limitations presents in this study. First, due to its cross-sectional design, the study cannot establish causal associations, and there remains the potential for confounding by unmeasured covariates. Second, the IS method merely indicated the theoretical effect of substituting one behavior for another, it may not fully encapsulate the complexity and dynamism of behavior changes in everyday life. Third, the use of accelerometers fails to capture certain types of activities (e.g., swimming, cycling) and the placement of the device (hipbone vs. wrist) may affect data accuracy. Fourth, depression and anxiety outcomes were self-reported. In spite of self-reported measures are more cost effective and convenient, there is a possibility of social expectation bias as respondents may conceal their true situation. Finally, since all the participants were restricted to three provinces in China, representation of the general population is limited. Future research should address these limitations to provide a more comprehensive understanding of the relationship between time-use compositions and mental health disorders in the university students.

## Conclusion

This study revealed that substituting 30 min of SB with LPA or MVPA significantly improved depression symptoms in university students. Greater benefits were observed when shifting SB to MVPA. Moreover, substituting 30 min of SB with MVPA was associated with reduced anxiety symptoms. These findings contribute valuable and novel information to our comprehension of how various intensities of PA impact mental health disorders. Future research should delve into the potential of PA as a cost-effective and readily accessible strategy to alleviate the burden of mental health disorders among university students.

### Electronic supplementary material

Below is the link to the electronic supplementary material.

Supplementary Material 1

## Data Availability

The datasets used and/or analyzed during the present study are available from the Y. Zhou (zhouyulan004@outlook.com) on reasonable request.

## Abbreviations

SB: Sedentary Behavior
LPA: Light-intensity Physical Activity
MVPA: Moderate-to-Vigorous Physical Activity
IS: Isotemporal Substitution
BMI: Body Mass Index
CES-D: Center for Epidemiologic Studies Depression Scale
SAS: Self-rating Anxiety Scale
CPM: Counts Per Minute
PSQI: Pittsburgh Sleep Quality Index
