# Supplementary material for: The effect of replacing sedentary behavior with different intensities of physical activity on depression and anxiety in Chinese university students: an isotemporal substitution model
Source: BMC Public Health. 2024 May 23;24:1388. doi: 10.1186/s12889-024-18914-y (PMC11118727; doi:10.1186/s12889-024-18914-y)
Supplement: Supplementary file 1 — Supplementary Material 1 [file 12889_2024_18914_MOESM1_ESM.docx]

Supplemental Table 1 Pearson’s bivariate correlations between SB, LPA, MVPA, and mental health disorders

|  | 1 | 2 | 3 | 4 |
| --- | --- | --- | --- | --- |
| 1. SB |  |  |  |  |
| 2. LPA | 0.166^*^ |  |  |  |
| 3. MVPA | −0.091 | 0.092 |  |  |
| 4. Depression score | 0.282^*^ | −0.088 | −0. 271^*^ |  |
| 5. Anxiety score | 0.259^*^ | 0.215^*^ | −0.362^*^ | 0.371^*^ |

Abbreviations: SB, Sedentary behavior; LPA, Light-intensity physical activity; MVPA, Moderate to vigorous physical activity.

*, *P* < 0.05.
